# BIOMARKERS HELP CLARIFY HOW SYSTEMIC AGING CONTRIBUTES TO MORTALITY: UTILIZING A MACHINE-LEARNING APPROACH IN HRS

**DOI:** 10.1093/geroni/igad104.0722

**Published:** 2023-12-21

**Authors:** Eric Klopack, Eileen Crimmins

**Affiliations:** University of Southern California, Los Angeles, California, United States; University of Southern California, Los Angeles, California, United States

## Abstract

Research suggests aging is a coordinated physiological decline occurring in multiple systems and at multiple biological levels. However, it is largely unknown how general biological aging and specific systemic aging co-occur and influence one another to affect mortality. There is also emerging interest in understanding how social exposures may differentially accelerate decline in individual physiological systems. We utilize data from the Health and Retirement Study, a nationally representative sample of about 4000 US adults over age 55. We used eXtreme Gradient Boosting (xgboost) in a training subsample to create estimates of mortality risk based on sets of biomarkers representing biological systems (e.g., brain and nervous system, adaptive immune system, cardiovascular system, renal system) as well as cellular aging (using epigenetic clocks and telomere length). These models generated mortality risk scores for each system (e.g., renal functioning using BUN, creatinine, and cystatin C). Mortality was regressed on these scores in a testing subsample to assess the relative impact of each system on mortality risk. Results suggest cellular aging, cardiovascular aging, and age-related metabolic dysfunction contribute most to mortality risk. Aging of these systems also partially mediates the mortality risk associated with age, race/ethnicity, sex, and education. These results suggest aging of specific systems may be differentially affected by sociodemographic exposures and may differentially contribute to mortality risk. This research may help guide interventions focused on reducing inequalities in mortality risk by identifying health risks that would have the greatest impact on mortality at the population level.

